# The Personality Traits and P300 of Offspring of Parents With Alcohol Dependence Differ Depending on Current Risky Drinking: A Preliminary Case-Control Study

**DOI:** 10.3389/fpsyt.2022.918965

**Published:** 2022-06-10

**Authors:** Yujia Qiu, Jing Wang, Ying Zhang, Tingfang Wu, Bing Li, Xin Yu

**Affiliations:** ^1^Clinical Research Department, National Clinical Research Center for Mental Disorders (Peking University Sixth Hospital), NHC Key Laboratory of Mental Health (Peking University), Peking University Institute of Mental Health (Sixth Hospital), Beijing, China; ^2^Ward Ten, Beijing Anding Hospital, Capital Medical University, Beijing, China

**Keywords:** alcohol dependence, offspring of parents with alcohol dependence, risky drinking, tridimensional personality, P300

## Abstract

**Aims:**

The aim of this study was to investigate the personality traits, and P300 component in the offspring of parents with alcohol dependence (OPAD) currently engaged in risky drinking and those not engaged in risky drinking, and to further explore the correlates of problematic alcohol use.

**Methods:**

A case-control study was conducted according to the cutoff of the Alcohol Use Disorder Identification Test (AUDIT). The frequency of the TaqIA polymorphism of the dopamine receptor D2 gene associated with alcohol dependence was compared between the two OPAD groups. Tridimensional Personality Questionnaire (TPQ), The Alcohol, Smoking, and Substance Involvement Screening Test (ASSIST), and the MINI-International Neuropsychiatric Interview (M.I.N.I.) were measured or interviewed in OPAD not engaged in risky drinking (resilient; *n* = 35) and those currently engaged in risky drinking (vulnerable; *n* = 20). P300 was measured to test the possible electrophysiological differences. The correlates of alcohol use were analyzed.

**Results:**

Vulnerable OPAD showed higher novelty seeking subscale scores (NS4; 4.45 ± 2.012 vs. 3.31 ± 1.728, *P* < 0.05) and harm avoidance subscale scores (HA4; 5.3 ± 2.319 vs. 3.66 ± 2.461, *P* < 0.05) than resilient OPAD, while the total scores of each dimension showed no significant difference. OPAD engaged in risky drinking showed more tobacco use than OPAD resistant to risky drinking. OPAD with risky drinking showed a shorter P300 latency than resilient OPAD on Fz electrodes. AUDIT scores of OPAD were correlated with P300 latency.

**Conclusions:**

P300 differed between OPAD with and without risky drinking and alcohol use was associated with P300 latency, indicating that P300 may be used in the early detection of vulnerable OPAD and early intervention in the future.

## Introduction

Alcohol dependence is a chronic mental disease that has both genetic and environmental determinants (1). Large numbers of offspring have parents with alcohol dependence, resulting from the high prevalence of alcohol dependence (2). Evidence shows that OPAD have a 2.5-to 4.4-fold increase in the possibility of developing risky drinking (3) and are more likely to have other negative social and mental outcomes (3–5). Causal associations between offspring drinking and parent alcohol use have even been reported (6, 7). In fact, similar to all other factors, parent alcohol use elicits a heterogeneous influence: some offspring remain resilient, but others indulge in drinking. However, studies on the specific characteristics differentiating vulnerable OPAD from resilient OPAD the differences are limited.

Genetic studies have shown that people with TaqIA of the dopamine receptor D2 gene (DRD2A1) is significantly associated with alcohol dependence (8). Many independent meta-analyses of alcohol dependence and controls have shown this association (9, 10). However, another study did not find an association between DRD2A1 and alcohol dependence (11). There was also evidence that the DRD2A1 was associated with alcohol use among young children of parents with alcohol dependence (12), while the difference in DRD2A1 between OPAD with and without risky drinking remains unclear.

Tridimensional personality, especially novelty seeking, differs between people with alcohol dependence and without alcohol dependence (13). Some studies have indicated novelty seeking personality traits as precursors of alcohol dependence (14, 15). Studies reported higher novelty seeking scores in the children of parents with alcohol dependence, but most used healthy controls for comparison (16, 17). However, there is still a lack of studies focusing on differentiating the personality trait between OPAD with and without risky drinking.

P300 event-related potential (ERP) has been shown to reflect an objective physiological basis of cognitive functions (18), such as attention-dependent information processing and stimulus categorization, which are impaired in a wide range of neurological and psychiatric disorders. The latency of the P300 is associated with stimulus evaluation time, reflecting processing speed (19), while the amplitude of the P300 is related to the intensity of processing (20). Previous evidence has indicated P300 as an endophenotype for alcohol dependence (21, 22). Some researchers found an abnormal amplitude of P300 during the No-Go condition in OPAD compared to control groups (23, 24). Evidence from functional magnetic resonance imaging (fMRI) studies has suggested that OPAD students with current alcohol problem showed greater activity of the middle frontal gyrus and reduced activation of the posterior cingulate in response to visual working memory and emotional processing tasks (25). However, the differences in P300 in OPAD with and without risky drinking are still obscure and require further investigation.

To better understand the biological factors that may be associated with the outcomes for OPAD, this study aimed to investigate the genetic, psychological and P300 characteristics in the OPAD currently engaged in risky drinking and those not engaged in risky drinking, and further explore the correlates of problematic alcohol use.

## Methods

### Participants

Fifty-five young adults were enrolled through advertisements on Wechat at Peking University Sixth Hospital. Participants completed the first screening to ensure whether they met the criteria for an offspring of a parent with alcohol dependence (OPAD) by asking “Did your father drink continuously for more than 1 year in your childhood and were there negative impacts on his physical and/or mental health, or an impact on his work or that of others?” according to the International Classification of Diseases, tenth edition (ICD-10) alcohol dependence criteria. Affirmations of all these three screening questions that indicated the participants were more than likely to be the offspring of parents with alcohol dependence. We only recruited offspring of fathers with ICD-10 alcohol dependence, in order to avoid the confounding effect of maternal alcohol abuse during pregnancy. All participants were right-handed and aged 18–45 years. They were excluded if they had a history of severe physical or neurological disease, conscious-loss or learning disability, or maternal alcohol use, and excluded if either parent had a history of schizophrenia, bipolar disorder or dementia.

All experimental procedures received approval from the Institutional Review Board of Peking University Sixth Hospital (No. 202046). All the participants provided written informed consent in writing at the beginning of the initial screening and participants were compensated for 100 RMB as their transportation allowance.

### Procedures

All participants were instructed to refrain from using any psychoactive substances, including alcohol, tobacco, caffeine, and sedative medications 24 h before testing. Each of them was interviewed and measured during three sessions, including gene sampling, psychological assessment, and EEG acquisition, which were all conducted by two well trained psychiatrists.

#### Genotyping

The 55 participants were instructed to clean their mouths using pure water and then provide oral swabs; DNA was extracted using standard techniques. DNA was used in the polymerase chain reaction as a template for the determination of DRD2 TaqI A alleles. DRD2 TaqI A genotyping was performed as described in another study (26). Psychiatrists who conducted the psychological assessment and ERP measurement were blinded to the identity of the samples. The A1A1 genotype was indicated by the uncleaned 310 bp fragment; the A1A2 genotype was indicated by three fragments: 310, 130, and 180 bp; and the A2A2 genotype was indicated by two fragments: 130 and 180 bp. There were eight A1A1, 25 A1A2 and 22 A2A2 genotypes. A dichotomous group variable (A1^+^ or A1^−^) was designated according to the genotype carried by participants. The A1^+^ group included eight A1A1 genotypes and 25 A1/A2 genotypes.

#### Assessment of Risky Drinking and Mental Health

The Alcohol Use Disorder Identification Test (AUDIT) was used to divide all the participants into the risky drinking and no risky drinking groups. Participants who met the criteria for the no risky drinking group had a score below 7 (*n* = 35) and those in the risky drinking group had score of 7 or greater (*n* = 20) according to the cutoff of 7 (27), which has been tested in China and found to be the best score to identify “risky drinking” in the Chinese population. The item-level content validity index was 0.83 and Cronbach's alpha was 0.782 (28).

Tridimensional Personality Questionnaire was a self-report instrument used to measure the personality tendency of OPAD, which included three dimensions (Novelty Seeking, NS; Harm Avoidance, HA; Reward Dependent, RD) and 12 subscales (NS1: exploratory excitability vs. rigidity; NS2: impulsiveness vs. reflection; NS3: extravagance vs. reserve; NS4: disorderliness vs. regimentation; HA1: anticipatory worry and pessimism vs. optimism; HA2: fear of uncertainty vs. confidence; HA3: shyness vs. gregariousness; HA4: fatigability and asthenia vs. vigor; RD1: sentimentality vs. insensitivity; RD2: persistence vs. irresoluteness; RD3: attachment vs. detachment; RD4 dependence vs. independence). TPQ is a normed and validated 100 item true-false questionnaire (29), and it has accepted validity and reliability in China (30). The Alcohol, Smoking, and Substance Involvement Screening Test (ASSIST) in this study was mainly used to assess other psychoactive substance use other than alcohol, such as tobacco, cannabis, cocaine, amphetamines, inhalants, sedatives, hallucinogens, opiates and other miscellaneous drugs. Concurrent validity was demonstrated by significant correlations between ASSIST scores (*r* = 0.59–0.88). The construct validity was 0.48–0.76 (29). The MINI-International Neuropsychiatric Interview (M.I.N.I.) were conducted in screening for mental disorders to rule out possible confounders. The criterion validity was ranged from 0.764 to 0.880, the concurrent validity within interviewers was 0.94 (*P* < 0.01), and the retest validity was 0.97 (*P* < 0.01) (30).

#### Auditory Oddball Paradigm

The paradigm consisted of frequently presented standard tones (90%), and infrequent target tones requiring a button press (10%). Standards (50 ms) and targets (100 ms) were 1,000 Hz, 75 dB pure tones. The task comprised a fixed pseudorandom sequence of 750 stimuli, with an interstimulus interval of 475–525 ms, divided into three blocks. Participants were instructed to press a response key to target tones only, using their right hand.

#### EEG Acquisition and P300 Extraction

Participants sat in front of a computer monitor and wore the headset for EEG recording during the oddball task (31). The EEG recording was digitized at 5,000 Hz from a 64-channel EEG system (Brain Products GmbH, Munich, Germany). The impedance of all electrodes was kept below 20 kΩ.

Signals were analyzed offline with the MATLAB R2014a (The Mathworks, Natick, MA, USA)-based EEGLAB toolbox (http://sccn.ucsd.edu/eeglab/). All recorded artifact-free EEG data were resampled to 500 Hz, referenced to an average of channels, and bandpass filtered in the range of 1–45 Hz to avoid the interference of 50 Hz signals. The time series were segmented into epochs with time- locked to the target auditory stimulus onsets (−200 to 500 ms) and baseline corrected (−200 to 0 ms). Epochs with excessive artifacts were removed. The data were then decomposed to perform an independent component analysis (ICA) *via* the runica algorithm. ICA components associated with vertical eye movements, heartbeats and other obvious artifacts were visually identified and removed according to their spatial, spectral, and temporal properties.

Participant ERP averages were calculated for target stimuli. P300 was identified as the most positive peak in a 235–400 ms window following stimulus onset at the Fz/Cz electrodes, where the P300 showed the largest amplitudes.

### Data Analysis

Demographic, personality and psychological health assessment, the amplitude and latency of P300 and the frequency of genotypes were analyzed with SPSS 26.0. All statistical tests were set at a significant level of *P* < 0.05. Categorical variables and continuous variables were compared between no risky drinking group and the risky drinking group by the chi-squared test, Wilcoxon signed-rank test and *t* test. We performed linear regression analysis to examine the relationships between AUDIT scores and other variables which showed different significantly between groups in the univariate analysis. These variables were used as independent variables in the linear regression analysis and the AUDIT scores were used as the dependent variable.

## Results

### Demographic Status, Clinical Feature, and Genotypes Among OPAD Based on Current Risky Drinking

The OPAD with risky drinking were similar in demographic status, including age, sex, ethnic group, areas where they were born, educational level, occupation, and marriage. With regard to clinical information, the risky drinking group showed higher scores on the AUDIT and ASSIST (tobacco) than the no risky drinking group. There was no significant difference in the genotype ratio between the two groups. No other psychoactive substances were used as measured by ASSIST (Table 1).

**Table 1:** Demographic status, clinical features and genotypes among offspring of parents with alcohol dependence based on current risky drinking.

	**No risky drinking** **(*N* = 35)**	**Risky drinking** **(*N* = 20)**	***t/Z/χ*** ^**2**^	***P*** **-Value**
**Demographic data**				
Age	28.8 ± 5.47	30.8 ± 5.45	−1.36	0.174
Sex (male/female)	6/29	8/12	3.504	0.061
Ethnic group (Han/other)	33/2	18/2	0.347	0.616
Born in city/country	27/8	18/2	1.414	0.297
Educational level	17.4 ± 2.64	17.8 ± 2.29	−0.019	0.985
Occupation (unstable/stable)	2/33	2/18	0.347	0.616
Marriage (not in marriage/in marriage)	25/10	13/6	0.053	0.817
**Clinical information**				
AUDIT score	0 (0, 2)	8 (7, 12)	−6.26	**<0.001**
ASSIT-tobacco	0 (0, 0)	3 (0, 14.75)	−3.174	**0.002**
M.I.N.I.				
Alcohol use disorder	0/35	4/16	7.549	**0.014**
Depression	15/20	13/7	2.497	0.097
Dysthymia	3/32	3/17	0.540	0.377
Mania	1/34	2/18	1.259	0.297
Panic disorder	1/34	1/19	0.167	0.599
Agoraphobia	2/33	1/19	0.013	0.703
Social phobia	2/33	2/18	0.347	0.616
Psychosis	2/33	2/18	0.347	0.616
Anorexia nervosa	1/34	3/17	2.783	0.131
Bulimia nervosa	1/34	4/16	4.526	0.053
Generalized anxiety disorder	2/33	5/15	4.262	0.086
Antisocial personality	0/35	1/19	1.782	0.364
**Genotype**				
A1+/A1–	22/13	11/9	0.327	0.567

*Data were presented as the mean ± SD, median (p25, p75) and ratio. P-values were obtained using the two-tailed two-sample t-tests for age and educational level, using two-tailed non-parametric test for AUDIT score and ASSIST (tobacco) score, and using two-tailed Chi-square test for other variables*.

*AUDIT, Alcohol Use Disorder Identification Test; ASSIT, Alcohol, Smoking, and Substance Involvement Screening Test; M.I.N.I., MINI-International Neuropsychiatric Interview*.

*Statistical significant values are shown in bold font*.

### Tridimensional Personality for Offspring of Parents With Alcohol Dependence Based on Current Risky Drinking

There were no significant differences in the total scores of novelty seeking, harm avoiding and reward dependence between the two groups. The risky drinking group of OPAD showed higher scores on NS4 and HA4 (uncorrected; Table 2).

**Table 2:** Personality of offspring of parents with alcohol dependence based on current risky drinking.

	**No risky drinking (*N* = 35)**	**Risky drinking (*N* = 20)**	***t*** **-Value**	***P*** **-Value**
Novelty seeking score	13.46 ± 4.468	15.05 ± 5.605	−1.027	0.304
Extravagance vs. reserve (NS1)	3.94 ± 1.662	4.2 ± 1.936	−0.792	0.428
Impulsiveness vs. reflection (NS2)	2.97 ± 1.843	2.85 ± 1.789	−0.621	0.534
Extravagance vs. reserve (NS3)	3.23 ± 1.784	3.75 ± 2.337	−0.742	0.458
Disorderliness vs. regimentation (NS4)	3.31 ± 1.728	4.45 ± 2.012	−1.986	**0.047**
Harm avoidance score	17.51 ± 6.464	19.65 ± 6.046	−0.991	0.322
Anticipatory worry/pessimism vs. optimism (HA1)	5.31 ± 2.398	5.75 ± 2.314	−0.732	0.464
Fear of uncertainty vs. confidence (HA2)	4.66 ± 1.454	4.65 ± 1.872	−0.428	0.668
Shyness vs. gregariousness (HA3)	3.89 ± 2.083	3.95 ± 2.139	−0.027	0.979
Fatigability and asthenia vs. vigor (HA4)	3.66 ± 2.461	5.3 ± 2.319	−2.292	**0.022**
Reward dependence score	17.38 ± 3.162	17.15 ± 3.2	−0.235	0.892
Sentimentality vs. insensitivity (RD1)	3.91 ± 1.055	3.8 ± 1.196	−0.226	0.821
Persistence vs. irresoluteness (RD2)	5.18 ± 1.866	4.9 ± 1.483	−0.732	0.464
Attachment vs. detachment (RD3)	6.41 ± 2.476	6.6 ± 2.415	−0.307	0.759
Dependence vs. independence (RD4)	1.88 ± 1.2	1.85 ± 1.268	−0.111	0.912

*Data were presented as the mean ± SD. P-values were obtained using the two-tailed two-sample t-tests for all the variables*.

*Statistical significant values are shown in bold font*.

### P300 for Offspring of Parents With Alcohol Dependence Based on Current Risky Drinking

**Figure 1:**
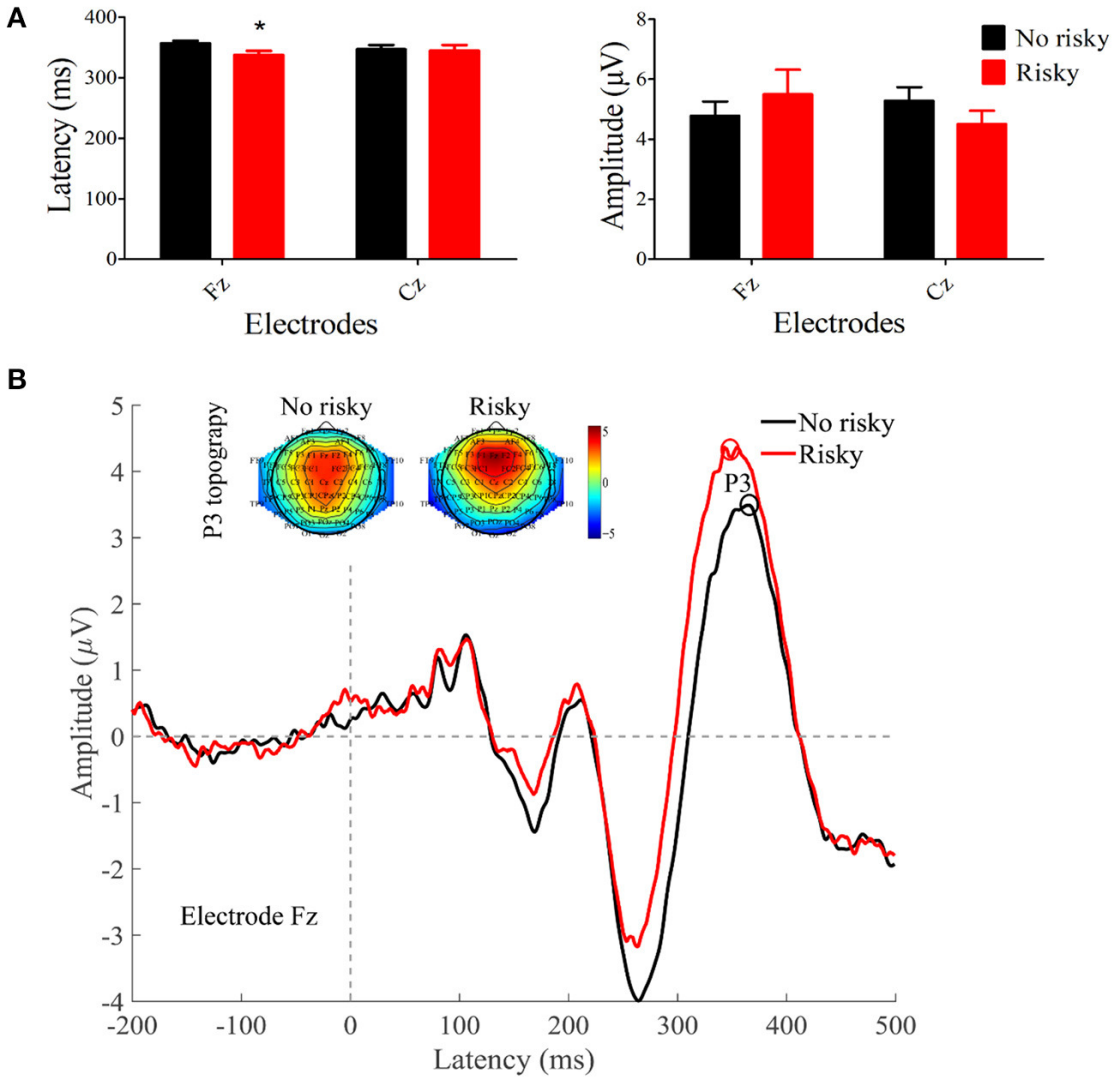
Comparison of P300 latency and amplitude between OPAD with and without risky drinking respectively. **(A)** Comparison of P300 latency and amplitude between two groups on electrodes of Fz and Cz; **(B)** Grand-averaged ERP waveforms in response to target stimuli in the first halves and the second halves as a function of group. Topographic maps of the P300 (235–400 ms) are shown. *P < 0.05.

Compared to OPAD without risky drinking, the latency of P300 at Fz in OPAD with risky drinking was shorter (*P* = 0.0153). There were no significant differences in the amplitude of P300 (Figure 1).

### Associations Between Risky Drinking and Psychophysical Characteristics

**Figure 2:**
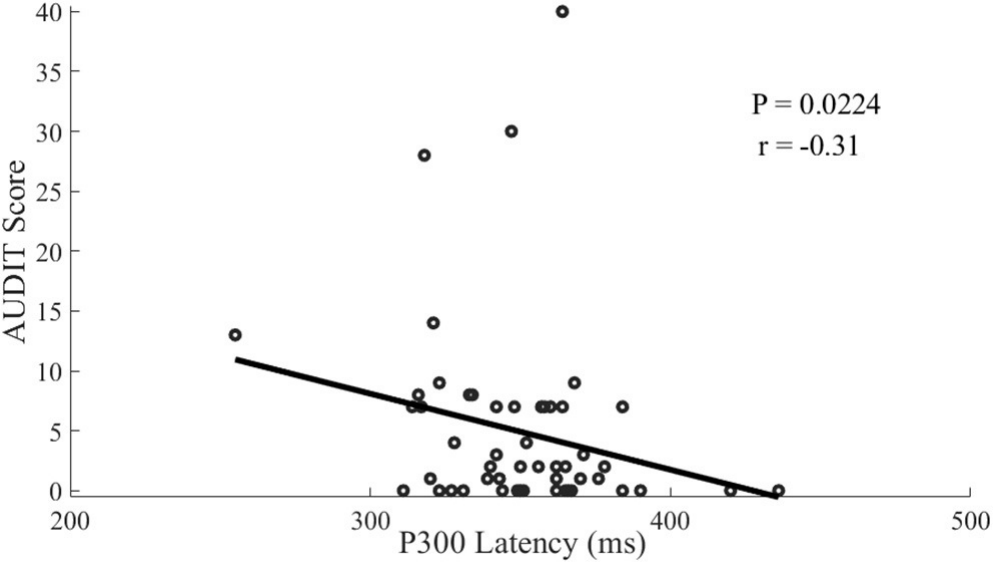
Scatterplot of the correlation between AUDIT scores and P300-lantancy among offspring of parents with alcohol dependence.

**Figure 3:**
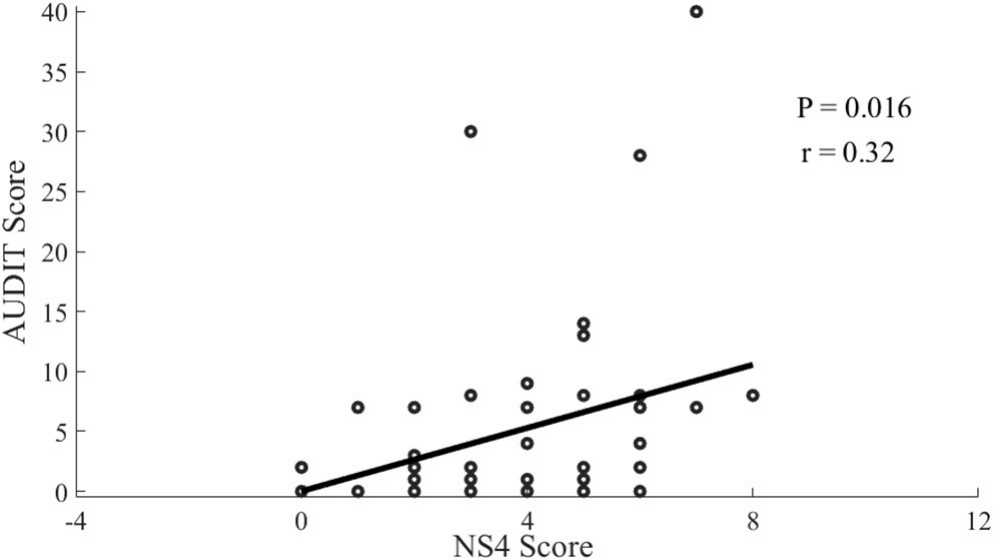
Scatterplot of the correlation between AUDIT scores and NS4 Score among offspring of parents with alcohol dependence.

**Figure 4:**
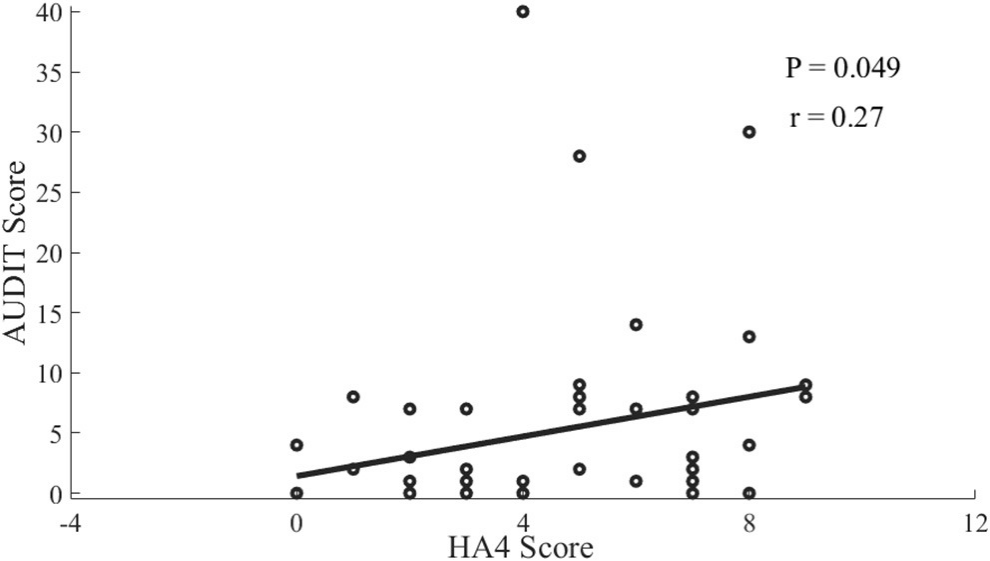
Scatterplot of the correlation between AUDIT scores and HA4 Score among offspring of parents with alcohol dependence.

The linear regression analysis showed that the AUDIT score was negatively associated (*r* = −0.31, *P* = 0.0224) with the latency of P300 at Fz electrode (Figure 2). In addition, AUDIT also showed a correlation with disorderliness vs. regimentation (NS4; *r* = 0.32, *P* = 0.016; Figure 3) and fatigability and asthenia vs. vigor (HA4; *r* = 0.27, *P* = 0.049; Figure 4).

## Discussion

In the current study, differences in personality and P300 were observed in OPAD currently engaged in risky drinking, compared with those without risky drinking. These differences included higher NS4 and HA4 scores, higher ASSIST (tobacco) scores and shorter latency of P300. Furthermore, the AUDIT scores were negatively correlated with the latency of P300 and positively correlated with NS4 and HA4 scores.

In this study, the total scores of novelty seeking, harm avoidance and reward dependence personality did not differ between OPAD with and without risky drinking, while OPAD with risky drinking showed more significant disorderliness (NS4) and fatigability and asthenia (HA4) than OPAD without risky drinking. The division of participants into groups based on their current risky drinking is justified by the observation that OPAD who do not have risky drinking at a young adult age are likely to represent resilient individuals, and OPAD with risky drinking are considered to be vulnerable individuals (25). Previous studies have showed that higher novelty seeking scores in the children of parents with alcohol dependence than in healthy controls (16, 17). Another study also found that novelty-seeking personality traits were more significant in families with high density of addiction than in those with a low density of addiction, and in unaffected people with positive family history of addiction, compared to people without positive family history (32–34). In a study investigating the three-dimensional personality between people with and without alcohol dependence, the former had a more apparent novelty-seeking personality than the control group (13). One study reported that compared with offspring of parents without alcohol dependence, OPAD had lower reward dependence scores and no significant difference in harm avoidance scores (17). Other studies showed that there were mixed results in comparing the differences of personality between unaffected people with and without family history of alcohol dependence (34, 35). The discordance may result from the different sample, study design, and assessment tools, in which the difference between groups among OPAD would not be as significant as the difference between probands and health controls, but it is meaningful to explore the possible precursor before the syndrome bursts out. However, the subtle higher NS4 and HA4 in OPAD with risky drinking can hint at the profile of these vulnerable people who were more disorderly and fatigable, which may help the clinician to understand and make more appropriate intervention strategies.

The risky drinking participants in the current study showed not only risky drinking but also significantly more tobacco use than the no risky drinking group. Prior studies also showed that both alcohol and other substances were used in OPAD, which may result from the need to reduce people's negative feelings (36, 37). Therefore, the current results indicated the possibility that OPAD with risky drinking may also use other substances to manage their possible emotional problems, which should be explored in the future.

This study showed the alteration in latency, not the amplitude of P300 in OPAD with risky drinking than in OPAD without risky drinking. Some previous studies found lower (24) or higher (38) amplitudes of P300 in OPAD than in healthy controls. Some reports did not find a significant difference in the latency of P300 between OPAD and healthy controls (21, 22). Our findings differ from those of other studies. This may be because all the subjects included in this study were OPAD and had no previously diagnosed psychological illnesses, which decreased the discrepancy while the comparison between OPAD and healthy controls would be more significant. As shorter P300 latency was associated with better information processing (39, 40), our different result may indicate that OPAD with risky drinking may process stimulus tasks faster than OPAD without risky drinking, which may be a compensatory mechanism of OPAD to risky drinking. The long-term outcome of the cognition under the impact of risky drinking should be further explored.

In our study there were significant negative correlations between AUDIT scores and P300 latency, while other studies didn't find such correlation (21, 22), possibly because other studies compared the OPAD with the healthy controls. The correlations in this study may result from that alcohol use at an early stage possibly increases the sensitivity and excitability of cerebral cortex (disinhibition), supported by the evidence of greater activity of middle frontal gyrus in young OPAD with risky drinking in response to visual working memory and emotional processing tasks (25). There were positive correlations between AUDIT scores and NS4 and HA4 in this study, which was not in accordance with other studies (16, 17), mainly due to different sampling as well. It indicated that the disorderliness and fatigability may increase the possibility of alcohol use among the OPAD, and vice versa, which can be explained by the mechanism of self-medication (36).

There are scarce studies focusing on vulnerability for offspring of parents with alcohol dependence and exploring possible psychophysiological mechanisms, especially controlling the genetic and other confounders. The results may provide a special and specific research direction for future studies in determining psychological and psychophysiological endophenotypes that can help with early warning and intervention. The current study further detected the difference between two relatively homogeneous groups of OPAD, and this should be more difficult in that both groups are already at risk and they are likely to be similar across many features. There are several possible limitations as follows. First, the probands were diagnosed according to the self-report of the OPAD, which may result in recalling bias, although we thoroughly checked the symptoms and criteria items to lessen the bias. Second, we do not include comparison groups with risky and no risky drinking whose parents have no history of alcohol use disorders, which may distinguish the impact from current risky drinking or the risk conferred from family. Selection bias may exist in that the participants were mostly had a high education level (as shown in Table 1) and stable occupation, which represented for higher socioeconomic status and may be protective factors. Further studies should recruit more representative participants from the general public. Another limitation of this study is the small sample size and unbalanced number of two groups, resulting in a more cautious explanation of the findings, because a small dataset is sensitive to deviation from the general population.

## Conclusions

In the current study, tridimensional personality and P300 latency may respectively distinguish offspring of parents with alcohol dependence based on their risky drinking and P300 latency was significantly correlated with AUDIT scores. Therefore, these findings may be used in the future early detection and intervention.

## Data Availability Statement

The original contributions presented in the study are included in the article/supplementary material, further inquiries can be directed to the corresponding author.

## Ethics Statement

The studies involving human participants were reviewed and approved by Institutional Review Board of Peking University Sixth Hospital. The patients/participants provided their written informed consent to participate in this study.

## Author Contributions

YQ and JW conducted the statistical analysis and drafted the original manuscript. XY conceived the study and revised the manuscript. YZ, TW, and BL collected data and reviewed the manuscript. All authors had final responsibility for the submission and all of them read and approved the final version of the manuscript.

## Funding

This study was supported by Self-exploration Project of National Clinical Research Center for Mental Disorders, Peking University Sixth Hospital (No. NCRC2020M10).

## Conflict of Interest

The authors declare that the research was conducted in the absence of any commercial or financial relationships that could be construed as a potential conflict of interest.

## Publisher's Note

All claims expressed in this article are solely those of the authors and do not necessarily represent those of their affiliated organizations, or those of the publisher, the editors and the reviewers. Any product that may be evaluated in this article, or claim that may be made by its manufacturer, is not guaranteed or endorsed by the publisher.
